# Study Protocol of tDCS Based Pain Modulation in Head and Neck Cancer Patients Under Chemoradiation Therapy Condition: An fNIRS-EEG Study

**DOI:** 10.3389/fnmol.2022.859988

**Published:** 2022-06-01

**Authors:** Brenda de Souza Moura, Xiao-Su Hu, Marcos F. DosSantos, Alexandre F. DaSilva

**Affiliations:** ^1^Headache & Orofacial Pain Effort (H.O.P.E.), Department of Biologic and Materials Sciences, School of Dentistry, University of Michigan, Ann Arbor, MI, United States; ^2^Laboratório de Propriedades Mecânicas e Biologia Celular (PropBio), Departamento de Prótese e Materiais Dentários, Faculdade de Odontologia, Universidade Federal do Rio de Janeiro (UFRJ), Rio de Janeiro, Brazil

**Keywords:** head and neck cancer, chemoradiotherapy, transcranial direct current stimulation, functional near-infrared spectroscopy, electroencephalograph

## Abstract

**Background:**

Multiple therapeutic strategies have been adopted to reduce pain, odynophagia, and oral mucositis in head and neck cancer patients. Among them, transcranial direct current stimulation (tDCS) represents a unique analgesic modality. However, the details of tDCS mechanisms in pain treatment are still unclear.

**Aims:**

(1) to study the analgesic effects of a protocol that encompassed supervised-remote and in-clinic tDCS sessions applied in head and neck patients undergoing chemoradiation therapy; (2) to explore the underlining brain mechanisms of such modulation process, using a novel protocol that combined functional near-infrared spectroscopy (fNIRS), and electroencephalograph (EEG), two distinct neuroimaging methods that bring information regarding changes in the hemodynamic as well as in the electrical activity of the brain, respectively.

**Methods:**

This proof-of-concept study was performed on two subjects. The study protocol included a 7-week-long tDCS stimulation procedure, a pre-tDCS baseline session, and two post-tDCS follow-up sessions. Two types of tDCS devices were used. One was used in the clinical setting and the other remotely. Brain imaging was obtained in weeks 1, 2, 5, 7, 8, and after 1 month.

**Results:**

The protocol implemented was safe and reliable. Preliminary results of the fNIRS analysis in weeks 2 and 7 showed a decrease in functional connections between the bilateral prefrontal cortex (PFC) and the primary sensory cortex (S1) (*p* < 0.05, FDR corrected). Changes in EEG power spectra were found in the PFC when comparing the seventh with the first week of tDCS.

**Conclusion:**

The protocol combining remote and in-clinic administered tDCS and integrated fNIRS and EEG to evaluate the brain activity is feasible. The preliminary results suggest that the mechanisms of tDCS in reducing the pain of head and neck cancer patients may be related to its effects on the connections between the S1 and the PFC.

## Introduction

Head and neck cancer affects annually more than 50.000 in the USA (Siegel et al., 2012). Pain is an important symptom reported by head and neck cancer patients. Therefore, the pathophysiology of cancer-related pain and the mechanisms of novel therapies used to ameliorate cancer pain must be explored in deep. It has been widely recognized that patients with locally advanced head and neck cancer undergoing definitive chemoradiotherapy (CRT) frequently experience severe pain due to the side effects related to cancer therapies. In this regard CRT has been combined with clinical guidelines such as symptomatic treatment and individualized pain medication, including opioids, to treat oral mucositis (OM) and tumor pain (Ling and Larsson, 2011; Elad et al., 2020).

In many cases, this leads to opioid overuse and, as a result, to drug-associated side effects, including tolerance, dependence, and addiction (Elting et al., 2008; Ling and Larsson, 2011; Schaller et al., 2015; Hu et al., 2016). Hence, it is imperative to elucidate not only the peripheral but also the central mechanisms associated with cancer-related pain. It is also necessary to explore the neuromechanisms by which different therapies are applied to ameliorate pain in cancer patients. Such information will be crucial to tailoring more specific therapies in a precision medicine context. This knowledge may help physicians improve the quality of life and reduce the side effects associated with CRT treatment.

Among the several novel adjuvant therapies that have been used in the treatment of cancer-related pain, the modulation of the neural activity of the primary motor cortex through transcranial direct current stimulation (M1-tDCS) has been proved to be a promising therapy. In fact, according to some preliminary results, tDCS can provide significant relief of pain in head and neck cancer patients under CRT treatment (Hu et al., 2016). However, the specific mechanisms by which tDCS acts to control cancer-related pain are still uncertain. Therefore, more studies adopting protocols that permit the evaluation of changes in the brain activity associated with tDCS must be developed to understand better its mechanisms in controlling cancer-related pain.

tDCS has been proven to be safe and very effective in treating different types of pain (Fregni et al., 2006; Dossantos et al., 2012; Donnell et al., 2015). Moreover, due to its safety aspects, it is a potential therapy for treating cancer pain. Due to its portability and easy handling, it would be reasonable to include tDCS as an additional tool in the palliative clinical setting. Supporting this concept, one case report demonstrated the feasibility and the benefits of tDCS therapy in patients with pancreatic cancer. According to the reported results, tDCS relieves pain and decreases the need for rescue medication (Silva et al., 2007). These effects of tDCS in the modulation of cancer-related pain may be at least in part explained by a significant electric current that flows not only through outer but also through inner cortical structures, as previously demonstrated by the so-called forward model analyses (Dasilva et al., 2012, 2015).

Nonetheless, other functional mechanisms must also be considered (Dossantos et al., 2016). In this regard, new devices that use several different configurations have been introduced in recent years. Such modifications of the original apparatus permitted researchers to study the effects of tDCS on brain activity using different neuroimaging methods, including electroencephalography (EEG) and functional near-infrared spectroscopy (fNIRS).

As a matter of fact, clinicians and researchers have sought objective pain assessment solutions *via* neuroimaging techniques for many years. They focused on the brain to detect how nociceptive signals and pain are processed in the human brain. Technological advances have made it possible to obtain responses to many old questions and to have more detailed information on the brain functioning under different conditions. In some cases, it is possible to extract real-time information about the brain activity during painful stimuli (Hu et al., 2019), which was unthinkable until some years ago. This accurate pain assessment is crucial across a wide range of acute and chronic pain conditions. It provides proper diagnosis and treatment, especially when patients have limitations in expressing their ongoing suffering. This is the case for many patients with head and neck cancer.

Multimodal integration, which combines multiple neurophysiological signals, has brought more attention in the last few years, primarily because of its potential to supplement a single modality's drawbacks and yield reliable results by extracting complementary features. One example is the integration of EEG with fNIRS which is cost-effective and, therefore, a fascinating approach to brain-computer interface (BCI) (Ahn and Jun, 2017; Hong et al., 2018; Ge et al., 2019). Overall, the integration of EEG and fNIRS provides us with two different sources of information about the brain, e.g., the electrical activities through EEG, and the hemodynamic responses, through fNIRS. This integration has the advantages of non-invasiveness, portability, and the previously mentioned cost-effectiveness (Ahn and Jun, 2017; Li et al., 2020a,b; Ghafoor et al., 2021).

More recently, simultaneous tDCS/EEG evaluation of cortical mechanisms provided information regarding the immediate effects of tDCS on the brain. Furthermore, an emerging technology called fNIRS has been used for brain imaging. fNIRS has become a reliable and objective tool to evaluate the cortical activity of patients by measuring changes in the blood oxygenation within different layers of the nervous tissue likewise functional magnetic resonance imaging (fMRI) (Schestatsky et al., 2013; Liang et al., 2014; Racek et al., 2015). Interestingly, a recent study reported the use of concurrent EEG/fNIRS to clarify hemodynamic changes in children that presented spasms in clusters (Bourel-Ponchel et al., 2017).

In our previous study, we applied tDCS pain neuromodulation in patients with head and neck cancer under CRT (Hu et al., 2016). We found that tDCS could offer significant symptoms relief for mucositis and odynophagia. Thereby, it helped reduce weight loss, improve performance status, and decrease narcotics intake. At the same time, tDCS induced the prefrontal cortex (PFC), the motor cortex (MC), and precuneus activations, as revealed by EEG data. However, the detailed brain mechanism was not well-understood at that time due to the limited number of EEG electrodes and the lack of spatial resolution of this method.

In the current study, we designed a new protocol that optimizes the one from our previous paper. More specifically, we added fNIRS to our study protocol. Also, we employed a tDCS device that can be administered remotely so that the tDCS sessions could be supervised and conducted in the patient's own place daily. fNIRS has become a reliable and objective tool to evaluate the cortical activity of patients by measuring changes in blood oxygenation, similar to fMRI (Ferrari and Quaresima, 2012; Curtin et al., 2019). The EEG/fNIRS combination has been proved to be effective in investigating the neurovascular coupling in the brain (Chiarelli et al., 2017; Pinti et al., 2021). On the other hand, the remotely supervised tDCS (RS-tDCS) has been an extension of in-clinic tDCS sessions that improve patients' compliance (Charvet et al., 2015; Kasschau et al., 2015; Shaw et al., 2017). Besides that, it has been shown that RS-tDCS represents an advance in the tDCS field, especially for patients with neurodegenerative diseases, including patients with multiple sclerosis and palliative care patients (Charvet et al., 2015; Kasschau et al., 2015, 2016; Shaw et al., 2017).

Hence, to provide a broader understanding of the central analgesic mechanisms linked to non-invasive neuromodulation in cancer-related pain, the protocol of the current study combined a 6-channel EEG with a 16-channel fNIRS system to investigate the effects of 20 sessions (remote and in clinic) of tDCS in head and neck cancer patients undergoing CRT.

## Methods

### Subjects

Patients with head and neck cancer undergoing definitive CRT treatment were recruited through the University of Michigan health system (UMHS), department of medical oncology. The CRT protocol included two Gray (Gy) per day, 5 days a week, for a total of 7 weeks. Subjects were screened by a group of clinical oncologists and further approached by the H.O.P.E. lab team members to discuss the study protocol and read the informed consent form.

The designed study protocol comprised a 7-week-long tDCS stimulation procedure, a pre-tDCS baseline session (pre-1 week), and two post-tDCS follow-up sessions (the first after 1 week and the second after 1 month). We used two types of tDCS devices. One of them was applied in our facility by our research staff, and the other one was a remotely supervised tDCS. The subjects visited our research facility every week. We used neuroimaging techniques to scan their brains during those sessions, which occurred in weeks 1, 2, 5, 7, 8, and after 1 month of tDCS treatment. We did two neuroimaging techniques in the current study, fNIRS and EEG.

This protocol (HUM00078942) was approved by the University of Michigan Institutional Review Board. Written informed consent was obtained from all participants.

### Inclusion Criteria

A. Subjects with head and neck malignancy were scheduled for CRT and were capable of understanding and adhering to the protocol requirements.
B. Subjects willing to comply with the study procedures and visits.
C. Subjects aged 18–75 years old.

### Exclusion Criteria

A. Substantial dementia.
B. Patients are being actively treated for another cancer at the time of enrollment.
C. Any condition that would prevent the use of tDCS and EEG, including any skull abnormality, implanted metals, implanted electronic devices, seizure disorders, neurologic conditions.
D. Use of an investigational drug or device within 30 days of study screening.

### Questionnaire

We used multiple questionnaires in the current study to evaluate patients' status along the study process. These questionnaires assessed pain, emotion, oral mucositis, weight, narcotic pain medication requirement, diet, and quality of life. A summary of the questionnaires used in this study can be found in Table 1.

**Table 1:** Questionnaires and clinical measurements in the current study.

**Assessment purpose**	**Questionnaire(s) used**
Pain	VAS, McGill, Geo-Pain
Emotion	PANAS-x
Oral mucositis	World Health Organization (WHO) scale, Oral Mucositis Weekly Questionnaire for Head and Neck Cancer
Weight	Clinical evaluation
Narcotic pain medication	Clinical evaluation
Diet	Clinical evaluation
Quality of Life	Head and Neck Quality of Life Weekly Questionnaire, University of Washington Quality of Life Questionnaire

In this proof-of-concept study, we recruited two participants. Both subjects received tDCS on the day of their CRT appointments. tDCS was applied before CRT when doing tDCS sessions in-clinic or after CRT when tDCS was self-administered at home. tDCS was applied daily (5 days per week) during the second and third weeks of CRT, three times per week during the fourth and fifth weeks of CRT, and twice per week during the sixth and seventh weeks of CRT (5/5/3/3/2/2 per week). The tDCS stimulation protocol was designed to accommodate the patient's CRT schedule in this study.

### In-clinic Procedures

The in-clinic tDCS protocol followed the methodology published in previous articles (Dasilva et al., 2011; Schestatsky et al., 2013). Briefly, the procedure was divided into seven steps: (1) check if all materials were available before starting the entire procedure. The checking items included the cap quality, the device battery, and the USB and Bluetooth connections between the device and the computer. Our study used C3 or C5 as references; (2) prepare the skin for tDCS stimulation. We inspected the skin for any pre-existing lesions—to avoid electrical stimulation/EEG recording over damaged skin or skull lesions. To increase conductance, we moved the hair away from the site of the electrical stimulation/EEG registering and placed plastic hair clips to keep hair away and cleaned the skin's surface to remove any signs of lotion, dirt, and grease. Furthermore, we allowed it to dry; (3) obtained patients' head measurements to decide the size of the cap in use. We marked the fiducial points, Cz, Fp1, and Fp2; (4) mount the electrodes onto the cap. We put conducting gel on the tDCS electrodes and embedded both EEG, and the tDCS electrodes into the cap; (5) patients wear the cap. We confirmed that the subject was seated comfortably and placed the cap in a way that the vertex of the cap matched the Cz point on the cap, while the Fp1 and Fp2 points were aligned as well. (6) configure the stimulation and data acquisition software (NeuroElectric, Spain) on the computer. (7) Finally, start the stimulation simultaneously with EEG data recording. We used a 2-mA current for the tDCS stimulation with a duration of 20 min per session.

### Remote Procedures

**Figure 1:**
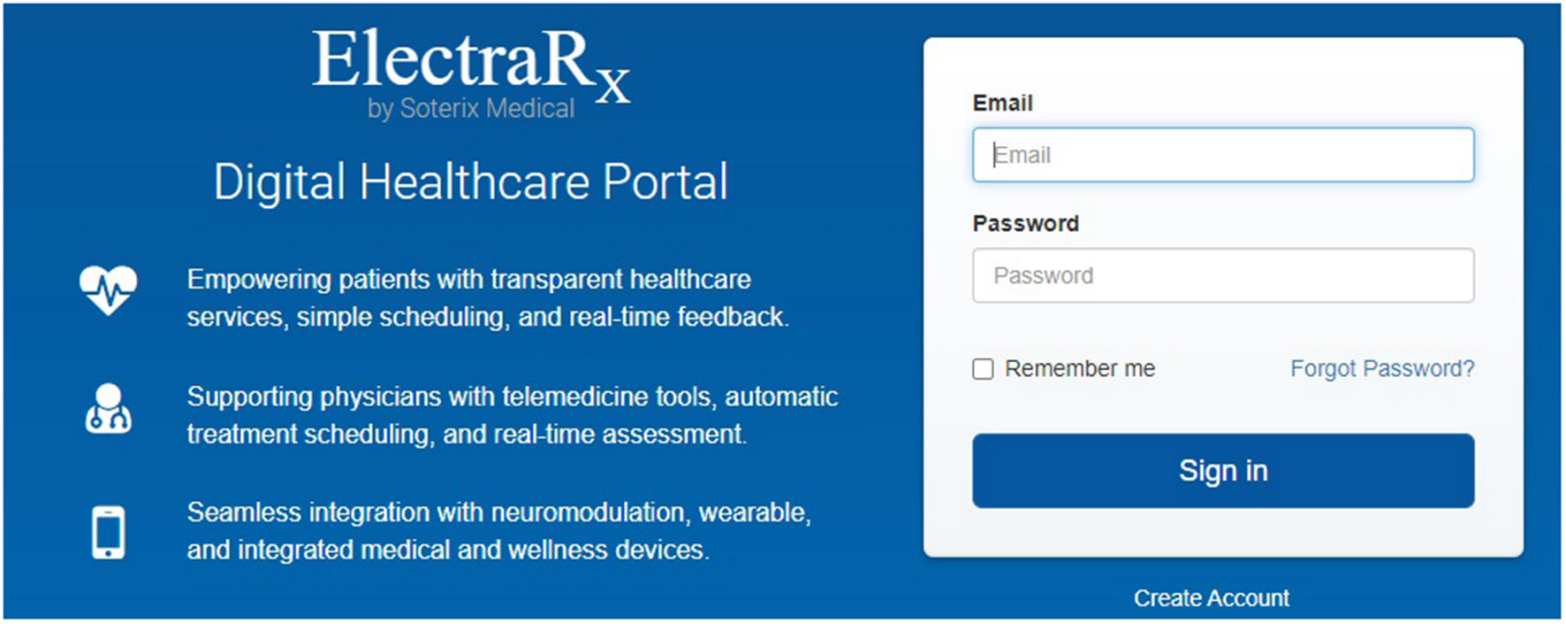
Remote tDCS website set-up. Further details can be found at: https://soterixmedical.com/research/remote/electrarx.

We used physical mini-CT tDCS devices (Soterix, NY) and online management software ElectraRx (Soterix, NY). At the end of the first in-clinic session, the patient received proper training on the remotely supervised tDCS using the ElectraRx website (https://www.soterixmedical.com/electrarx/login) and was provided with a mini-CT device and written guidelines. Then the patients were able to complete their stimulation sessions at home. In each remote session, the patients filled out the steps through the ElectraRx website ahead of the remote session start and then were provided with a code that allowed the participant to start the stimulation (Figure 1). We used a 2-mA current for the tDCS stimulation with a duration of 20 min per session.

Patients were asked to properly follow the steps to ensure correct electrode preparation and placement, low impedance, and safe device removal. During the remote sessions, the patients were instructed to abort the RS-tDCS session in case of any significant discomfort or other adverse events, or if a study team member determined that the session should be discontinued. Also, they were aware of the designated “stop criteria.” If the stop criteria were met at any time throughout the study, the session and/or ongoing study participation were reviewed.

Finally, the subjects completed a “tDCS side effects form” after each stimulation. This assessment objectively gauged any adverse events that the patient underwent as a direct result of the stimulation.

### Neuroimaging

**Figure 2:**
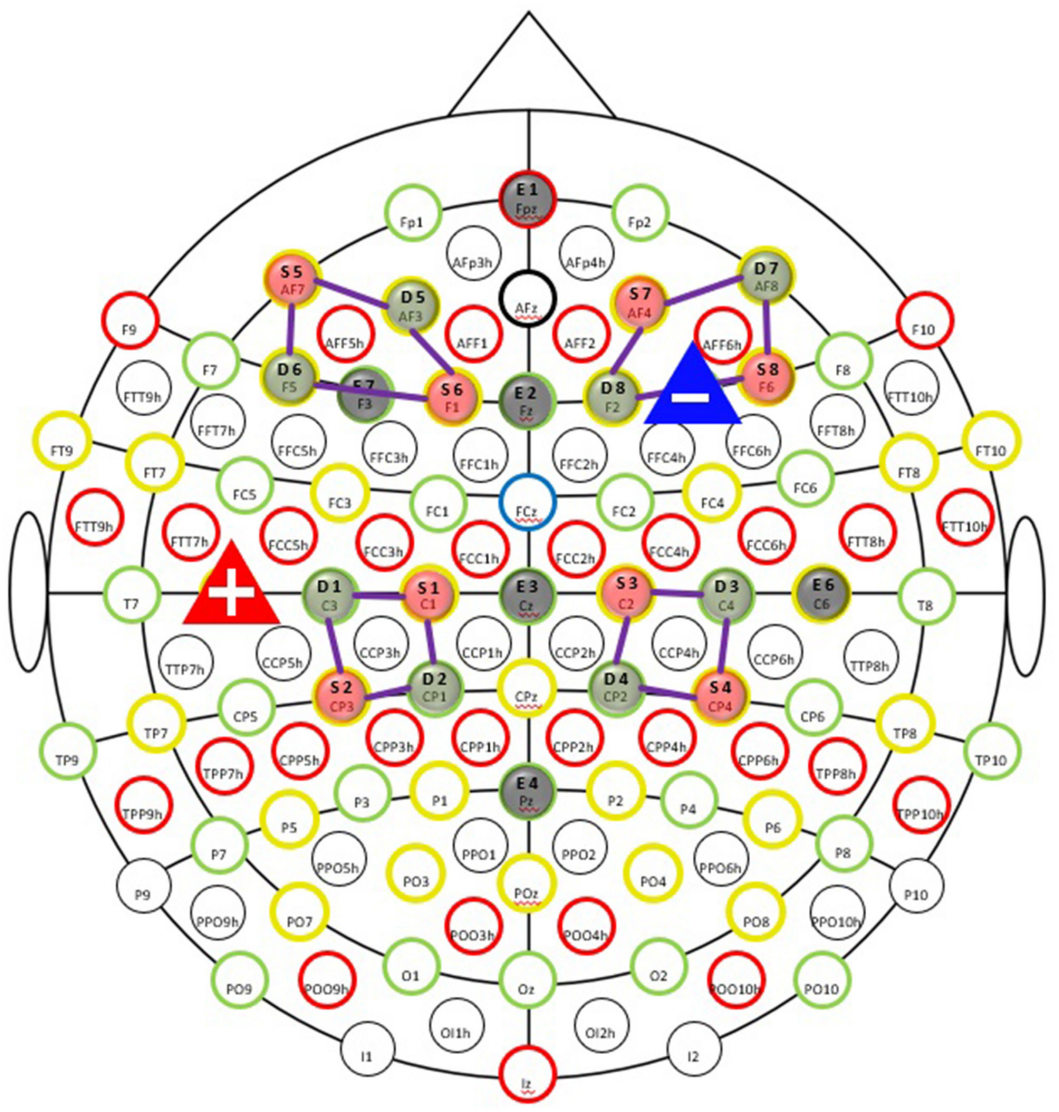
Simultaneous EEG-fNIRS-tDCS multimodal scanning and stimulation setup EEG evaluation of cortical mechanisms can elucidate valuable information regarding the immediate tDCS effects on the brain. EEG recording was taked at the pre-study visit, the first stimulation visit, last stimulation on first week of tDCS session, and the fifth and seventh week of treatment (both on the last session on week), as well as the follow-up appointment. The fNIRS is an important tool for clinical monitoring of tissue oxygenation and measurement of cortical activity, thereby appear that an advancement in brain imaging. fNIRS was taked at the pre-study visit, first stimulation visit, at the fifth week of treatment and the last stimulation visit.

We used an NE EEG/tDCS device (Neutralelectrics, Spain) combined with NIRx fNIRS (NIRx Medical Technologies, Germany) device for the neuroimaging data collection. The montage for the EEG/fNIRS/tDCS probes can be found in Figure 2. Our neuroimaging probe cap design was based on the international 10-10 system reference map. The Table 2 provides the relevant fiducial markers in the 10-10 system and MNI coordinates (Koessler et al., 2009). We placed the tDCS anode at the right F4 fiducial mark, while the cathode was placed at the left C5 fiducial mark. Then we placed the EEG electrodes, respectively, at Fpz, Fz, Cz, Pz, F3, and C6 fiducial marks, yielding 6 EEG data channels. Our EEG data were collected at a sample rate of 500 Hz. Finally, our fNIRS setup employed 8 by 8 source-detector combinations (3 cm apart), yielding 16 data measurement channels. The data channels, respectively, covered the bilateral prefrontal cortices and bilateral somatosensory/motor cortices. Our fNIRS data were sampled at a rate of 7.81 Hz. The localization process was validated *via* a photogrammetry-based localization process (Hu et al., 2020).

**Table 2:** The neuroimaging localization results for fNIRS optodes, channels, EEG, and tDCS electrodes.

**Type**	**Modality**	**10-10 location**		**MNI coordinates**	
			**X**	**Y**	**Z**
Source 1	fNIRS	C1	−25.1	−22.5	70.1
Source 2	fNIRS	CP3	−46.9	−47.7	49.7
Source 3	fNIRS	C2	26.7	−20.9	69.5
Source 4	fNIRS	CP4	49.5	−45.5	50.7
Source 5	fNIRS	AF7	−41.7	52.8	11.3
Source 6	fNIRS	F1	−22.1	26.8	54.9
Source 7	fNIRS	AF4	35.1	50.1	31.1
Source 8	fNIRS	F6	52.9	28.7	25.2
Detector 1	fNIRS	C3	−49.1	−20.7	53.2
Detector 2	fNIRS	CP1	−24	−49.1	66.1
Detector 3	fNIRS	C4	50.3	−18.8	53
Detector 4	fNIRS	CP2	25.8	−47.1	66
Detector 5	fNIRS	AF3	−32.7	48.4	32.8
Detector 6	fNIRS	F5	−51.4	26.7	24.7
Detector 7	fNIRS	AF8	43.9	52.7	9.3
Detector 8	fNIRS	F2	23.6	28.2	55.6
Electrode 1	EEG	Fpz	1.4	65.1	11.3
Electrode 2	EEG	Fz	0	26.8	60.6
Electrode 3	EEG	Cz	0.8	−21.9	77.4
Electrode 4	EEG	Pz	0.7	−69.3	56.9
Electrode 5	EEG	F3	−39.7	25.3	44.7
Electrode 6	EEG	C6	65.2	−18	26.4
Anode	tDCS	F4	41.9	27.5	43.9
Cathode	tDCS	C5	−63.6	−18.9	25.8
**Type**	**Modality**	**Estimated brain region**		**MNI coordinates**	
	**Source-detector**		**X**	**Y**	**Z**
Channel 1	fNIRS S1-D1	BA^*^ 6	−37.1	−21.6	61.65
Channel 2	fNIRS S1-D2	BA 2|BA 4|BA 1|BA 6|BA 3	−24.55	−35.8	68.1
Channel 3	fNIRS S2-D1	BA 3|BA 4|BA 6|BA 1|BA 2	−48	−34.2	51.45
Channel 4	fNIRS S2-D2	BA 5|BA 2|BA 1|BA 3	−35.45	−48.4	57.9
Channel 5	fNIRS S3-D3	BA 6	38.5	−19.85	61.25
Channel 6	fNIRS S3-D4	BA 6|BA 1|BA 2|BA 4|BA 3	26.25	−34	67.75
Channel 7	fNIRS S4-D3	BA 3|BA4|BA 1|BA 2|BA 6	49.9	−32.15	51.85
Channel 8	fNIRS S4-D4	BA 1|BA 2|BA 3	37.65	−46.3	58.35
Channel 9	fNIRS S5-D5	BA 9|BA 8	−37.2	50.6	22.05
Channel 10	fNIRS S5-D6	BA 9|BA 46|BA 8|BA 10	−46.55	39.75	18
Channel 11	fNIRS S6-D5	BA 8	−27.4	37.6	43.85
Channel 12	fNIRS S6-D6	BA 8|BA 6	−36.75	26.75	39.8
Channel 13	fNIRS S7-D7	BA 9|BA 10	39.5	51.4	20.2
Channel 14	fNIRS S7-D8	BA 8	29.35	39.15	43.35
Channel 15	fNIRS S8-D7	BA 46|BA 9|BA 10	48.4	40.7	17.25
Channel 16	fNIRS S8-D8	BA 8	38.25	28.45	40.4

* *BA stands for Brodmann Area*.

### Optodes and Electrodes Registration

We designed the cap in three sizes, 56, 58, and 60, respectively, to account for head size variation, following the international 10-10 transcranial system positioning. We then applied a photogrammetry method to register all the optodes and data channels onto the cortical surface. Our previous article described the detailed method (Hu et al., 2020). Briefly, we used the Structure Sensor (Occipital Inc, CO) with an iPad (Apple Inc, CA) to capture the 3D photos of the optodes and electrodes on the participants' heads. We then loaded the 3-D photo in the MATLAB software (Mathworks, MA, USA) and pinpointed the locations of fNIRS optodes and EEG/tDCS electrodes with five fiducial markers (Nasion, Inion, Cz, AR, and AL in the 10-10 system). The derived optodes coordinates were affinely transferred into the MNI space using the MATLAB-based AtlasViewerGUI toolbox (Aasted et al., 2015). The mid-points between the source-detector (optodes) pairs were used as the coordinates for the fNIRS channels. Finally, we estimated the regions under each fNIRS channel and EEG electrode by matching their MNI coordinates with locations in the neurosynth.org database. Also, we evaluated the brain regions with a voxel size of 10 mm using the WFU_pick atlas in XJview toolbox (https://www.alivelearn.net/xjview).

### Pain Level Evaluation

**Figure 3:**
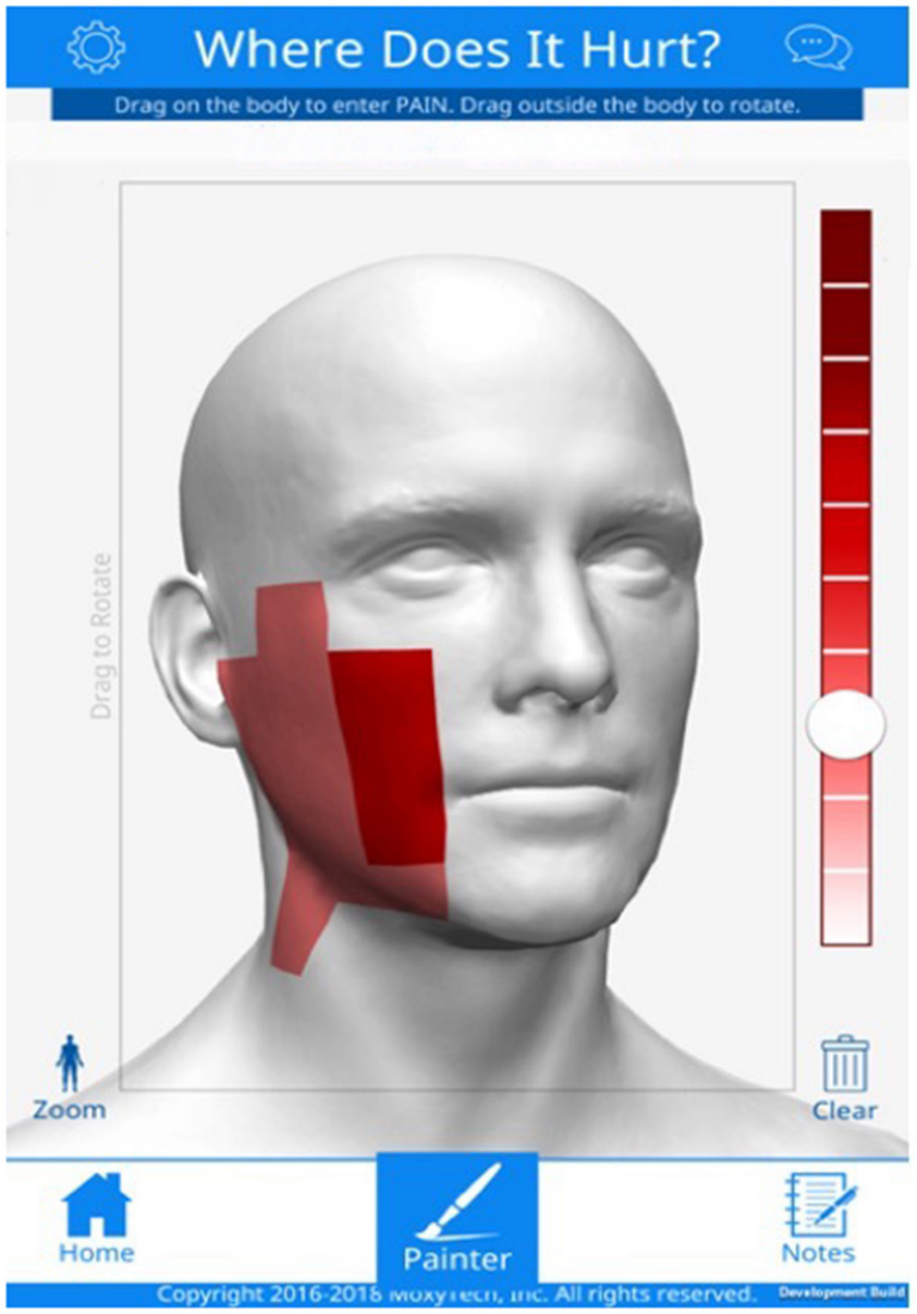
Geo-pain software graphical user interface, MoxyTech Inc, MI.

Patients' pain levels were evaluated immediately before and after the stimulation through the McGill Pain Questionnaire (MPQ)—Short Form (Melzack, 1975) and through the GeoPain App (Kaciroti et al., 2020). GeoPain is a free stand-alone and embedded mobile app developed in collaboration with the Headache and Orofacial Pain Effort (HOPE) at the University of Michigan and is currently licensed by the spinoff MoxyTech Inc (Kaciroti et al., 2020). GeoPain provides a 3D body map for pain tracking based on a squared grid system with vertical and horizontal coordinates using anatomical landmarks (an example can be found in Figure 3).

### Neuroimaging Data Analysis

fNIRS and EEG data collected in the current study were analyzed separately and then interpreted jointly. The fNIRS data were analyzed using the NIRSAnalyzer toolbox (Santosa et al., 2018), while the EEG data were analyzed using the EEGLAB toolbox (Delorme and Makeig, 2004) in MATLAB (Mathworks, MA).

We conducted functional connectivity analysis on the collected fNIRS data to study the brain mechanism induced by tDCS. The calculation process was described in a previous method paper (Santosa et al., 2017). Briefly, the raw fNIRS data was first downsampled to 4 Hz. Then we converted the raw data into oxy- and deoxy-hemoglobin using the modified Beer-Lambert law (Kocsis et al., 2006). We then used bandpass filters to filter that HbO data into two frequency bands, namely, high (0.5–1 Hz) and low (0.01–0.08) frequency bands. These two frequency bands were selected to avoid the physiological signal bandwidth, including Mayer's wave (0.1 Hz), respiratory (0.3–0.5 Hz), and cardiac (1–1.5 Hz) relevant fluctuations. Next, we calculated the between-channel correlation at the individual level using the connectivity method in the toolbox. This method used prewhitening *via* the autoregressive filter and the weighted least-squares fit during the FC calculation to account for physiological noises, motion, and other artifacts in the fNIRS signal (Santosa et al., 2017). Then, the individual-level correlation coefficients were converted to Z-score using Fisher's Z-transform. Finally, a linear mixed-effect (LME) model was applied to obtain the group-level connectivity effect. We applied the LME analysis using the MixedEffectsConnectivity function in the NIRSAnalyzer toolbox. Briefly, the LME estimated the group-level correlation effects based on the individual *Z* scores. The model consists of two parts, fixed effects and random effects. We used the individual subject as random effects to account for between-subject variability so that the fixed effects can be estimated as the group-level correlation effects. This calculation was implemented on the data collected from both subjects during the two lab visits. It is worth noting that we included a relatively high-frequency band (0.5–1 Hz) connectivity analysis in this study for exploratory EEG-fNIRS network comparison on the delta frequency band (0–4 Hz).

For EEG data, we conducted a dipole source localization analysis on the collected EEG signal. First, we visually inspected the EEG data to reject artifact-affected segments. The inspected EEG signals were first filtered using a bandpass filter with cut-off frequencies at 0.01–40 Hz. Then, the data was cleaned using the automatic data cleaning pipeline in the EEGLab toolbox and re-referenced. We ran an independent component analysis (ICA) on the preprocessed signal. The calculated independent components were then entered into the DIPFIT plug-in in the EEGLAB toolbox for the source localization (Kavanagh et al., 1978; Oostendorp and Van Oosterom, 1989). The head model used for the DIPFIT was the BEM DIPFIT head model with MNI coordinates. In addition, we used the phase-lag index (PLI) to analyze the functional connectivity between the electrodes. The PLI measures the phase synchronization of EEG signals recorded from different electrodes [CITATION]. In this study, we calculated the PLI using the following equation:


(1) $$PLI=|\frac{\sum_{t=1}^{K}sign(Im[e^{-i{\left(\theta^{x}-\theta^{y}\right)}_{t}}])}{K}|$$

Where K is the number of the phase differences, *t* is the time from 1 to *K, x* and *y* are, respectively, the electrode indices, and *sign* is the sign function. The PLI value ranges from 0 to 1, where 0 means inconsistent phase lag (volume conduction), while 1 means perfectly consistent phase lag.

Finally, the fNIRS and EEG analysis results were gathered for a qualitative and quantitative comparison and correlation. For qualitative comparison, we plotted the possible tDCS induced brain mechanism in a network consisting of (1) cortico-cortical connections revealed by the fNIRS signal, (2) cortico-deep-brain connections revealed by the EEG signal, if available. Also, the collected questionnaire data and preprocessed neuroimaging data were entered into a canonical correlation analysis model for brain-behavior correlation change in EEG power spectra. For quantitative analysis, we calculated the correlation between the fNIRS connectivity at low/high-frequency bands and the EEG power spectral density at different frequencies (respectively, at 4/8/16/24 Hz). The Pearson's and Spearman's correlation coefficients were calculated, and the relative *p*-values were examined to reveal the potential relationship between the fNIRS signal and EEG signal.

### Preliminary Results

**Figure 4:**
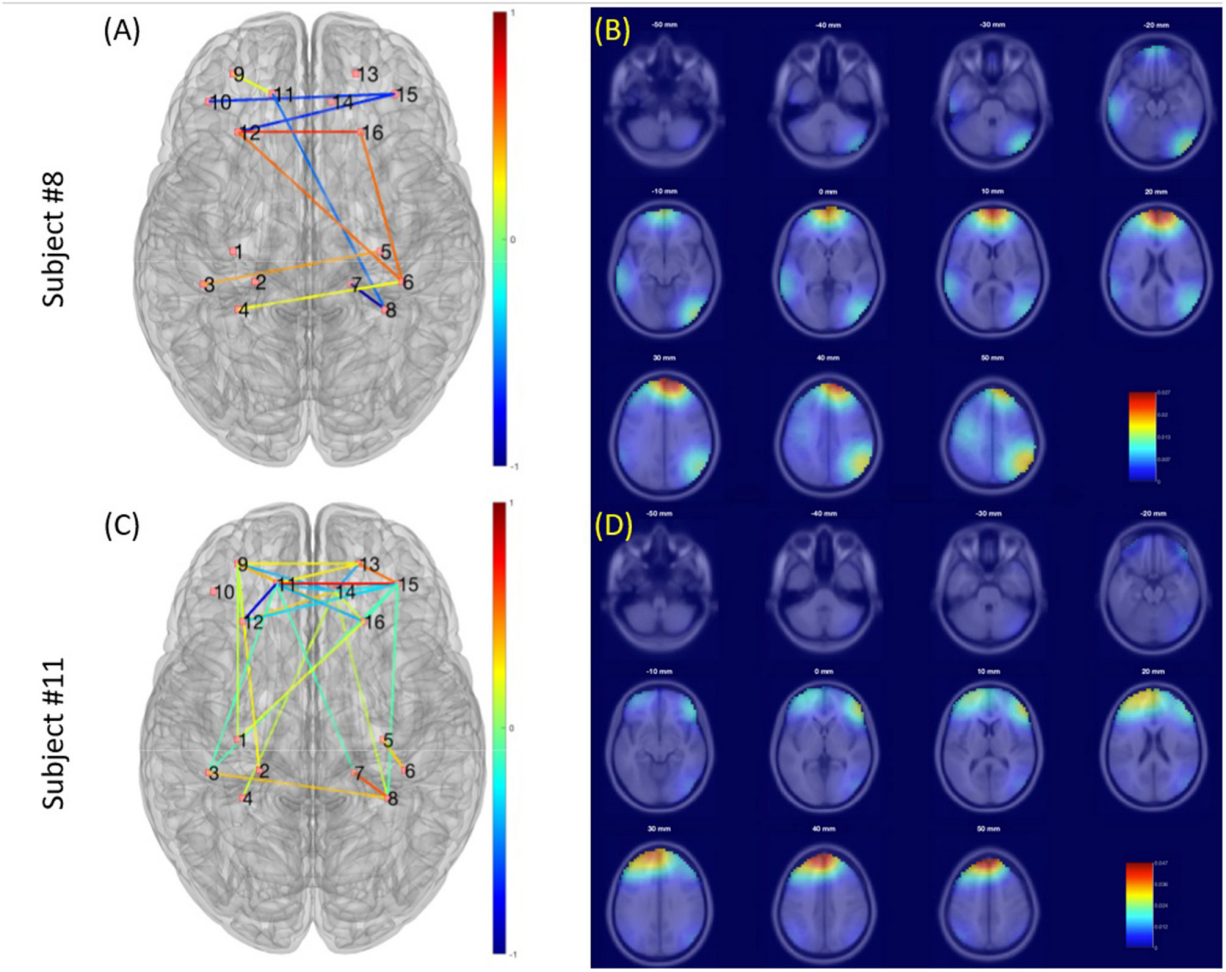
Brain activations during tDCS stimulation (Week 2, 5, and 7 combined). **(A,C)** Functional connectivity revealed by fNIRS. **(B,D)** Source localization of EEG signal.

**Figure 5:**
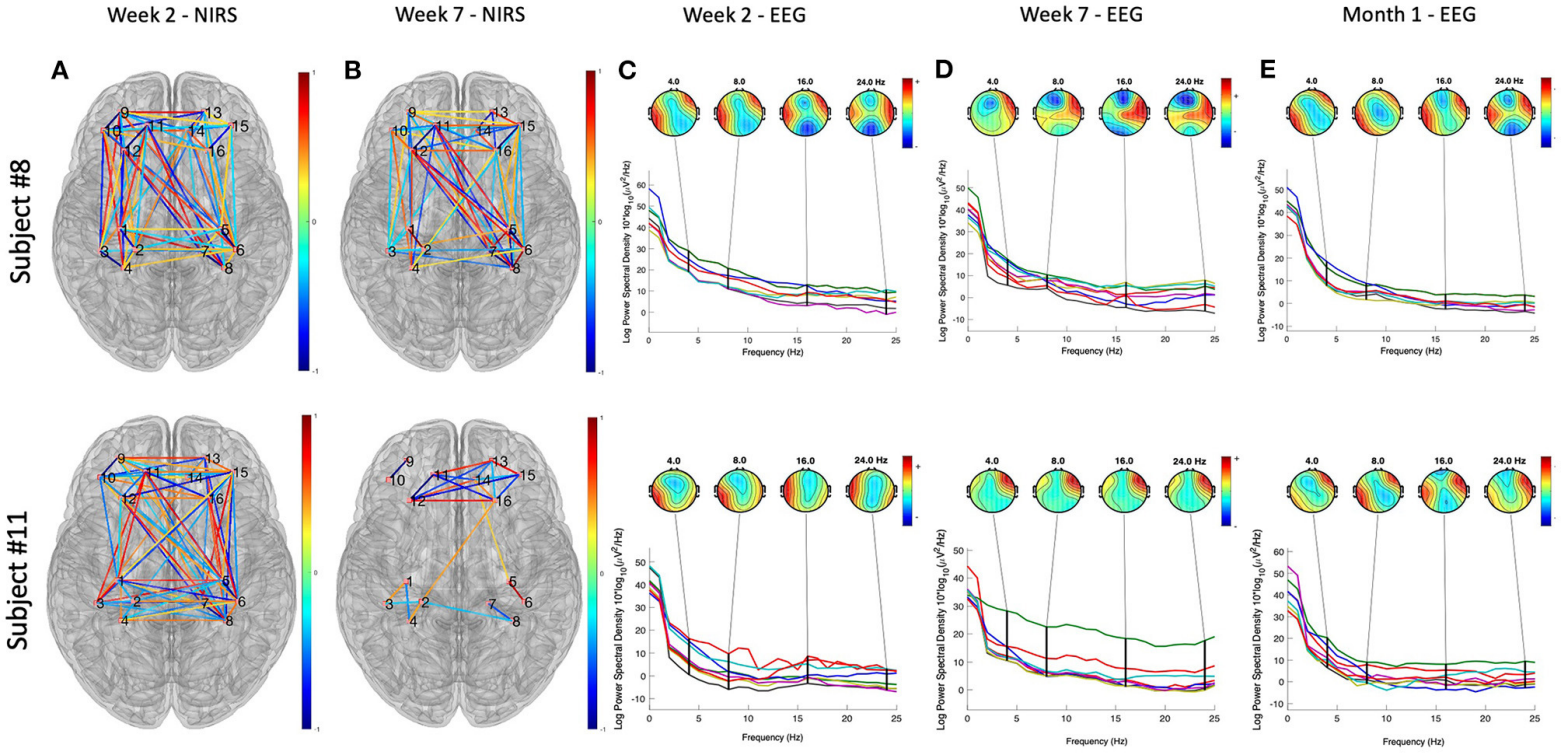
Neuroimaging data analysis results of the resting state before tDCS stimulation at week 2, week 7, and 1-month follow-up, respectively, for subjects #8 and #11. **(A,B)** Show the significant connections between bilateral prefrontal and sensory cortices revealed by the fNIRS data (*p* < 0.05, FDR corrected) in week 2 and week 7. The color bar represent the correlation coefficient levels, ranges from −1 to 1. **(C–E)** Demonstrate the power spectra map of EEG data, separately at 4/8/16/24 Hz, in week 2, week 7, and month 1 follow-up. The color bars represent the power of the spectral density at different frequencies, ranges from 0 to 60.

The data presented in this section were collected from two subjects who have participated in the study. We first presented the demographic and questionnaire data in Table 1. Figure 4 illustrates the results of neuroimaging data analysis during tDCS stimulation, respectively, for Subjects #8 and #11. Panels (a) and (c) present the significant connections between bilateral prefrontal and sensory cortices revealed by fNIRS data (*p* < 0.05, FDR corrected). Differences in EEG power spectra in the PFC were found when comparing the seventh with the first week of tDCS. Panels (b) and (d) show the results of the EEG source localization analysis, presented as a transverse view at different slicing. Figure 5 shows the connectivity patterns of the resting state before tDCS stimulation at week 2, week 7, and one-month follow-up, respectively, for subjects #8 and #11. Panels (a) and (b) show the significant connections between bilateral prefrontal and sensory cortices revealed by the fNIRS data (p < 0.05, FDR corrected) in week 2 and week 7. Panel (c), (d), and (e) demonstrate the power map of EEG data at 4/8/16/24 Hz, respectively, in week 2, week 7, and month 1 follow-up for subjects #8 and #11.

## Discussion

The current study presented a preliminary protocol that applies tDCS modulation of pain in head and neck cancer patients under CRT. Furthermore, patient-bedside neuroimaging techniques, e.g., fNIRS and EEG, were used to monitor the associated underlining brain mechanisms. tDCS was able to decrease the functional connections between the PFC and S1 according to the fNIRS data. The EEG data revealed that the 7 weeks of tDCS activated the right PFC compared to the first week of stimulation (week 2).

The collected visual analog scale (VAS) score and amount of narcotic use (Table 3) indicated that both patients suffered from the worst pain in the weeks 5–week 8 along the CRT treatment process. Such severe pain was probably due to the side effects of the CRT treatment since both patients presented little pain and no narcotic use at the beginning of the treatment. We then analyzed the resting state data collected as baseline data before tDCS sessions in weeks 2, 7, and 1-month follow-up. The EEG power maps at different frequency bands (4/8/16/24 Hz) demonstrated a similar pattern of right prefrontal cortical activation after 7 weeks of tDCS. fNIRS data obtained from both participants demonstrated less PFC-S1 connection after 7 weeks of tDCS stimulation. In addition, we found that both participants developed PFC activation during the tDCS modulation, as revealed by the EEG signal. Participant # 8 also demonstrated right parietal activation, while participant # 11 demonstrated some very weak activation in bilateral parietal cortices. Previous studies have confirmed that the PFC, including the anterior PFC and the dorsal lateral prefrontal cortex (DLPFC), play a key role in pain perception (Peng et al., 2018). Our observations revealed by the preliminary data also indicated that the PFC was activated during the tDCS modulation (Figure 5). Also, the PFC decreased in connection with the motor and sensory parts of the brain, as indicated in Figure 5, panels (a) and (b). We also found that the 7-week tDCS modulation protocol increased the broadband (4/8/16/24 Hz) EEG power at the right PFC region in both the subjects enrolled in this study. While in Subject # 8, we observed a decrease in the activation in the anterior PFC after 7-week stimulation, consistent with the findings of a previous study (Roy et al., 2014). Unfortunately, we have not collected data from a standard of care (control) group to make further conclusions.

**Table 3:** Preliminary demographic and clinical measurement data collected from two patients.

	**VAS**	**VAS**	**pre-PANASx**	**pre-PANASx**	**Narcotic**	**Weight**	**Mucositis**	**QoL WA**	**QoL**	**PPI**
	**(0–5)**	**(0–100)**	**-PA**	**-NA**	**_use**			**pain gen**	**H&N**	**_Mcgill**
**Subject # 8 Age = 62 Gender = M**										
Wk0	0	0			0	276	0	20	0	0
Wk1	0	0	29	24	0	276	0			0
Wk2	3	40	27	25	0	272	1			0
Wk3	2	20	30	21	0	260	4			2
Wk4	3	25	20	21	0	257	0			
Wk5	2	40	25	20	25	239	3			3
Wk6	2	80	24	23	300	242	3			
Wk7	2	20	23	23	420	240	2	30	0	2
Wk8	3	60	24	25	840	240	4	30	5	
Mo1	1	30	34	23	630	232	4	30	1	2
**Subject # 11 Age = 60 Gender = M**										
Wk0	0	0			0	211		10	1	0
Wk1	0	0	36	10	0	211				0
Wk2	2	20	43	10	0	212				5
Wk3	4	60	49	10	0	208	4			2
Wk4	2	20	46	10	70	208	3			3
Wk5	1	10	42	10	70	209	1			2
Wk6	2	20	44	10	140	203	4			4
Wk7	3	70	44	10	140	204	4	40	3	2
Wk8	3	40	43	10	140	208	3	40	1	3
M	3	40	43	10	0	208	3	30	1	2

*PANAS, Positive and Negative Affect Schedule; PA, Positive Affects; NA, Negative Affexts; QoL, Quality of Life; WA, Washington; H&N, Head and Neck; PPI, Present Pain Intensity*.

During the experiment protocol designed and data collection, we experienced several challenges that presented limitations to the study. The first challenge in this study is designing a probe holding cap that holds both EEG electrodes and fNIRS optodes. The two neuroimaging techniques required placement of their probes on the head surface, and thus the space for each probe could be very limited (Chiarelli et al., 2017). The cap design included two regions of interest, the PFC and the S1. Therefore, we used the F7, F8, C3, and C4, in the international 10-20 system as the centers to deploy fNIRS/EEG probes. Specifically, we used four fNIRS optodes (two sources and two detectors, forming up 4 channels) to surround one EEG electrode, as illustrated in Figure 4. In addition, the number of EEG electrodes was not sufficient. The electrodes employed as anode/cathode for tDCS stimulation cannot record EEG signals. Therefore, we only had 6 electrodes for EEG signal recording, which may lead to less-optimal source localization analysis for EEG data.

Moreover, the fNIRS and EEG signal analysis do not have a standard pipeline (Pinti et al., 2021). The advantage of the dual-modality measurement was to detect both hemodynamic responses and neurophysiology signals using an entire portable setup. fNIRS has balanced spatial and temporal resolutions. However, it detects only the cortical response. Thus, EEG is a supplementary imaging technique not only to measure neuronal signals that couples hemodynamic responses but also from both cortical and deep brain regions. We combined the two imaging techniques and investigated both cortico-cortical and cortico-deep brain networks.

Nonetheless, the joint data analysis is challenging due to the neurovascular coupling (Huneau et al., 2015; Phillips et al., 2016). Previous studies analyzed the two types of data separately and then correlated the preprocessed signals (Pinti et al., 2021). Recently, a novel study recorded EEG signal as the stimulus function for the hemodynamic response modeling and then used the model to fit the fNIRS data from different channels (Zhang and Zhu, 2019). By fitting the hemodynamic response model to the fNIRS data, future studies will be able to obtain both cortico-cortical and cortico-deep-brain connections based on the two types of signals.

Finally, the large amount of clinical data collected through the questionnaires pose challenges on clinical information-brain correlation. Such correlation is a key step that associates the collected neuroimage data with the clinical data acquired from the questionnaires and clinical measurements. Along with the protocol in the current study, we collected eight categories of clinical measures or questionnaires along with the study at multiple time points. On the other hand, we collected fNIRS data from 16 data channels, 3 sessions (75 min in total), and EEG data from 6 channels, 5 sessions (125 min in total) from each participant. The large amount of data posed challenges to associating neuroimaging with clinical measures. Existing studies proved that methods like canonical correlation analysis (CCA) (Wang et al., 2020) or artificial intelligence (machine learning) analysis (Mihalik et al., 2020) could be used to solve such “big data” correlation issues. In the current study, we condensed the neuroimaging data by finding the connectivity between the brain regions of interest. In future studies, we will use a method like CCA or un-supervised artificial intelligence algorithms to find precisely which “clinical score” correlates mainly with the specific “connection” revealed by the neuroimaging data analysis.

Our understanding of tDCS-based head and neck pain modulation is very limited. In this article, we proposed a novel protocol to study (1) the effect of tDCS-based pain modulation (remote-supervised and in-clinic) in head and neck patients undergoing CRT and (2) the underlining brain mechanisms of such modulation process. The study results will enhance understanding of the mechanisms by which tDCS modulates cancer-related pain. We also expect that our study and data will represent a starting point for more complicated protocols of tDCS-based cancer-pain modulation.

## Data Availability Statement

The original contributions presented in the study are included in the article/supplementary material, further inquiries can be directed to the corresponding authors.

## Ethics Statement

The studies involving human participants were reviewed and approved by University of Michigan Institutional Review Board. The patients/participants provided their written informed consent to participate in this study.

## Author Contributions

AD conceived the project. BS collected the data. X-SH analyzed the data. BS, X-SH, MD, and AD led the writing. All authors read and accepted the final version of the manuscript. All authors contributed to the article and approved the submitted version.

## Funding

We thank the MCubed Award–University of Michigan and the Brazilian Government Agencies: CNPQ (Conselho Nacional de Desenvolvimento Científico e Tecnológico), Fundação de Amparo à Pesquisa do Estado do Rio de Janeiro (FAPERJ), for their financial support. This research did not receive any grants from funding agencies in the commercial sectors.

## Conflict of Interest

AD is the co-creator of GeoPain and cofounder of MoxyTech Inc, which licensed the technology from the University of Michigan. The remaining authors declare that the research was conducted in the absence of any commercial or financial relationships that could be construed as a potential conflict of interest.

## Publisher's Note

All claims expressed in this article are solely those of the authors and do not necessarily represent those of their affiliated organizations, or those of the publisher, the editors and the reviewers. Any product that may be evaluated in this article, or claim that may be made by its manufacturer, is not guaranteed or endorsed by the publisher.
